# Bilateral Serous Psammocarcinoma of Ovary: Rare Variant Low Grade Serous Carcinoma

**DOI:** 10.1155/2015/531242

**Published:** 2015-10-18

**Authors:** Saubhagya Kumar Jena, Pritinanda Mishra, Vandana Mohapatra, Sweta Singh

**Affiliations:** ^1^Department of Obstetrics & Gynaecology, All India Institute of Medical Sciences (AIIMS), Bhubaneswar, Bhubaneswar, Odisha 751019, India; ^2^Department of Pathology and Laboratory Medicine, All India Institute of Medical Sciences (AIIMS), Bhubaneswar, Bhubaneswar, Odisha 751019, India

## Abstract

Serous psammocarcinoma is a rare variant of serous carcinoma arising from either ovary or peritoneum, characterized by massive psammoma body formation, low grade cytologic features, and invasiveness. Its clinical behavior is similar to serous borderline tumors with relatively favorable prognosis. We report herein a case of a 60-year-old postmenopausal woman who presented with abdominal distension. Contrast enhanced computed tomography (CECT) revealed calcified pelvic masses with ascites. Elevated serum CA-125 (970 U/mL) suggested malignant ovarian neoplasm. Patient underwent exploratory laparotomy with primary debulking surgery. Histopathology showed bilateral serous psammocarcinoma of ovary with invasive implants on omentum. Adjuvant chemotherapy was advised in view of advanced stage disease, although its benefits are poorly defined due to rarity of the tumor. However, patient opted out of it and is now on follow-up.

## 1. Introduction

Serous psammocarcinoma is a rare variant of serous carcinoma arising either from ovary or peritoneum [1]. It is characterized by extensive formation of psammoma bodies, invasion of ovarian stroma, peritoneum or intraperitoneal viscera, and moderate cytological atypia. It has relatively better prognosis compared to serous carcinoma [1]. Thorough MEDLINE search revealed 34 cases of ovarian psammocarcinoma reported till date. We describe here a case of bilateral serous psammocarcinoma of ovary in a 60-year-old Indian woman.

## 2. Case Report

**Figure 1:**
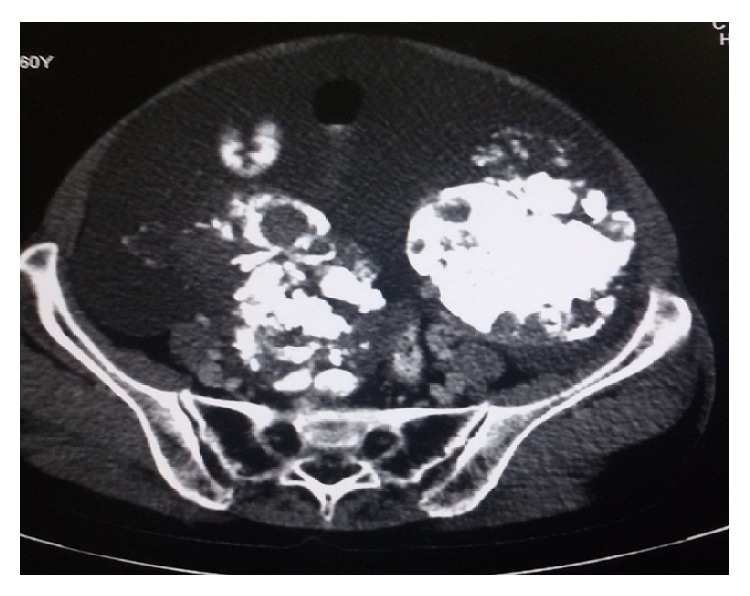
CECT image showing bilateral calcified pelvic masses.

A 60-year old postmenopausal woman presented with complaints of abdominal distension and discomfort of four-month duration. On examination, two separate solid, irregular, and fixed abdominopelvic masses were palpable along with evidence of ascites. The masses were felt separate from uterus per vaginum. Bilateral breast examination was normal. Ultrasonography showed complex, calcified abdominopelvic masses in both iliac fossae along with ascites and mild omental thickening. Contrast enhanced computed tomography (CECT) revealed ascites and calcified pelvic masses measuring 12 × 9 × 8 cm^3^ on left side and 12 × 8 × 8 cm^3^ on the right (Figure 1). Serum Cancer Antigen-125 (CA-125) was elevated (970 U/mL). These findings suggested possibility of malignant ovarian neoplasm. However, cytology of peritoneal fluid obtained under ultrasound guidance was negative for malignant cells. Upper GI endoscopy was normal.

**Figure 2:**
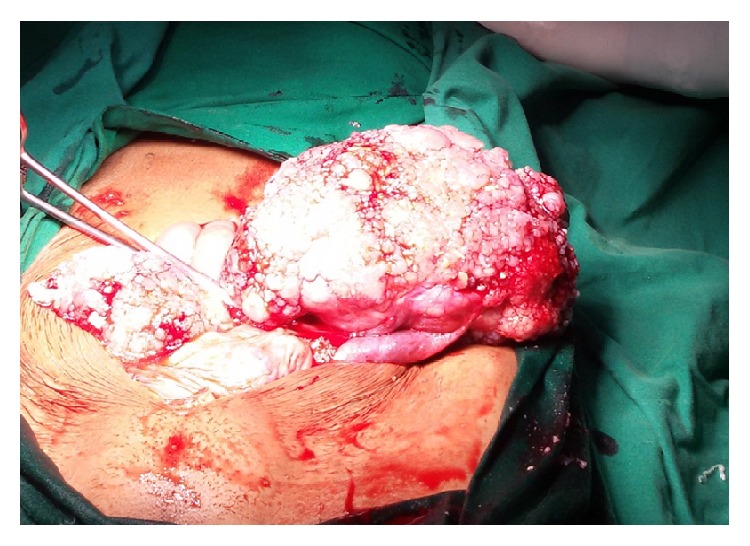
Intraoperative images showing bilateral ovarian masses with diffuse calcification.

On exploratory laparotomy, 5 litres of ascites fluid was drained. Bilateral ovaries were enlarged with tumor nodules on surface (Figure 2). Peritoneum over sigmoid colon and omentum was studded with tumor deposits of size < 1 cm. Total abdominal hysterectomy with bilateral salpingo-oophorectomy was performed along with infracolic omentectomy and resection of peritoneal lesions. No enlarged retroperitoneal lymph nodes were palpated during surgery, so lymphadenectomy was not done. Her postoperative recovery was uneventful.

**Figure 3:**
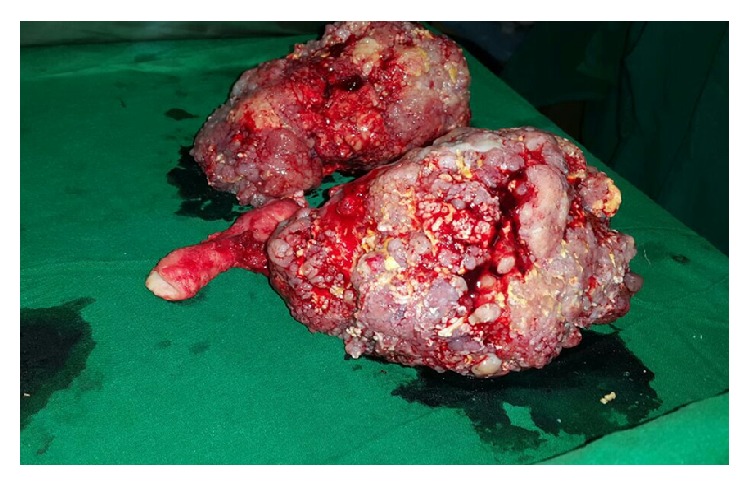
Postoperative image of the specimen.

**Figure 4:**
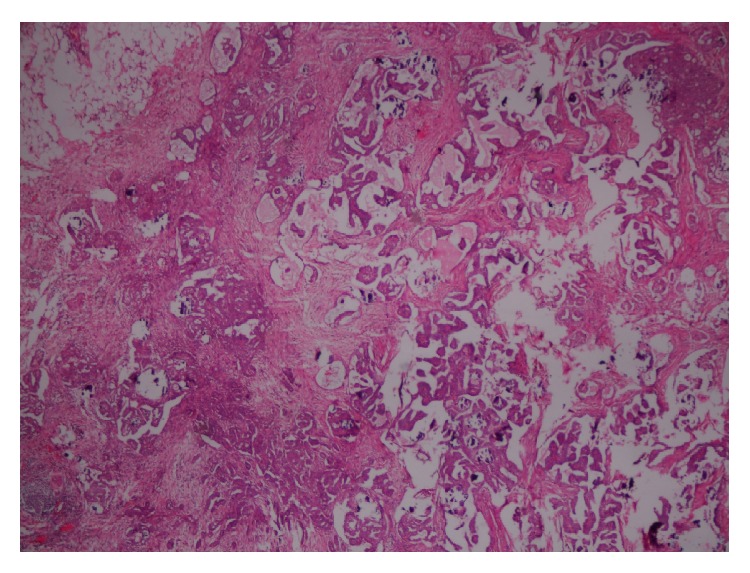
Peritoneum showing invasive implants (H&E 100x).

**Figure 5:**
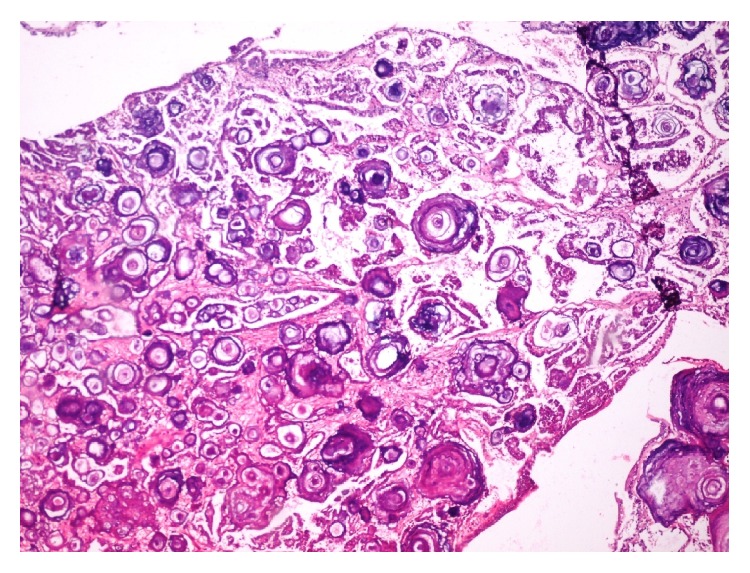
Microscopic picture showing extensive psammoma body formation (H&E 100x).

Grossly, bilateral ovarian surface was bosselated with breach in capsule. Both ovaries were enlarged with left ovary measuring 14 × 11 × 10 cm^3^ and right ovary measuring 14 × 10 × 8 cm^3^ (Figure 3). Cut section was predominantly solid with variegated areas and more than 50% of the tumor showed gritty yellowish areas of calcification. Histopathology showed low grade serous psammocarcinoma of bilateral ovaries with invasive implants on omentum and peritoneum, putting her at International Federation of Gynecology and Obstetrics (FIGO) Ovarian Cancer Stage IIIB (Figure 4). There was moderate degree of pleomorphism with scanty mitotic figures [2/10 HPF] (Figure 5). In view of advanced stage disease, adjuvant chemotherapy with paclitaxel and carboplatin was advised. However, patient opted out of it. She is now on follow-up for the past one year after surgery. No recurrence was evident radiologically and CA-125 levels remained normal during follow-up.

## 3. Discussion

Serous psammocarcinoma was first described by Grimaldi et al. in 1916 [2]. Gilks et al. in 1990 proposed the following histologic criteria for diagnosis of psammocarcinoma: destructive invasion of ovarian stroma or intraperitoneal viscera; no more than moderate cytologic atypia; no areas of solid epithelial proliferation except for occasional nesting: no more than 15 cells in diameter; and at least 75% of papillae associated with or completely replaced by psammoma body formation [1]. According to these criteria, our case was diagnosed with primary ovarian serous psammocarcinoma. Extensive literature search showed presence of only 65 cases of psammocarcinoma of ovary and peritoneum reported till date; out of that, 31 were primary peritoneal and 34 primary ovarian cases [3, 4].

Psammoma bodies are multiple, discrete, and laminated calcified bodies formed by accumulation of calcium on single necrotic or degenerated tumor cells. Psammoma bodies have rarely been demonstrated in cervicovaginal Pap smears and in cytology of peritoneal washing in cases of ovarian psammocarcinoma [5, 6]. Histopathologic differential diagnosis of psammocarcinoma involves other borderline serous epithelial tumors, cystadenofibromas, and adenocarcinomas. It can be differentiated from them by Gilks et al. criteria [1, 7, 8].

Psammocarcinoma of ovary is characterized by low proliferative activity with Ki67 and diploid DNA [9]. Activating mutations in BRAF which occur in low grade serous neoplasms have been identified in these tumors [10].

Clinically, patients usually present with abdominal discomfort and increase in abdominal girth. These tumors are usually unilateral, but rarely may these be bilateral as in our case [11]. CA-125 levels are commonly elevated [12]. Computed tomography (CT) scan findings of psammocarcinoma are extensively calcified pelvic and peritoneal masses [13]. The pelvic mass appears sandy and coarsely granular on enhanced T1 weighted magnetic resonance images due to scattered clusters of psammomatous calcification [13]. Fluorodeoxyglucose positron emission tomography/computed tomography (FDG PET/CT) may have advantage over conventional CT in differentiating malignant calcifications from benign ones based on glucose metabolic activity [14].

Psammocarcinoma has been suggested to have more favourable prognosis compared to invasive serous adenocarcinoma with low recurrence following tumor resection [1]. Although most seem to follow an indolent course similar to that of borderline tumors of the ovary, rarely has aggressive clinical behavior with distant metastasis and recurrence been reported [15, 16].

Because of the rarity of this tumor, there are no standard treatment guidelines [17]. Most authors recommend aggressive cytoreduction as initial treatment [17]. Patients have been treated with adjuvant or neoadjuvant chemotherapy in the past, but its benefits are poorly defined. However, recurrence of disease has been reported when no adjuvant chemotherapy was given [7]. Recently, Chase et al. observed excellent clinical and biological response following postoperative chemotherapy in a case of advanced ovarian psammocarcinoma [16]. They suggested optimal debulking followed by adjuvant chemotherapy as the standard treatment for advanced stage disease. Rare aggressive forms reported in the past raise doubts on the supposed indolent course of this tumor and warrant close follow-up.

We report this case of bilateral serous psammocarcinoma of ovary as it is a rare variant of serous carcinoma of ovary, usually associated with better prognosis in advanced stages also. Our case was also in advanced stage but there is no recurrence of disease either clinically or radiologically without chemotherapy. Her serum CA-125 level also decreased and is presently within normal range. Although the standard treatment of ovarian psammocarcinoma is surgery followed by few cycles of chemotherapy in the aggressive forms, we currently lack evidence of increasing the benefits that can bring chemotherapy in the management of advanced ovarian psammocarcinomas. Future trials in wide yard are required to establish the role of postoperative chemotherapy in such cases.
